# Assessing Causality in Associations of Serum Calcium and Magnesium Levels With Heart Failure: A Two-Sample Mendelian Randomization Study

**DOI:** 10.3389/fgene.2019.01069

**Published:** 2019-10-23

**Authors:** Emilie Helte, Agneta Åkesson, Susanna C. Larsson

**Affiliations:** ^1 ^Unit of Nutritional and Cardiovascular Epidemiology, Institute of Environmental Medicine, Karolinska Institutet, Stockholm, Sweden; ^2 ^Department of Surgical Sciences, Uppsala University, Uppsala, Sweden

**Keywords:** calcium, heart failure, magnesium, minerals, Mendelian randomization

## Abstract

Evidence from observational studies suggests that increased exposure to calcium may increase the risk of coronary heart disease and stroke whereas magnesium might have a protective effect on disease risk. However, studies of the associations of these minerals with heart failure are scarce and limited by potential biases introduced by confounding and reverse causality. We applied a two-sample Mendelian randomization design using summary estimates to assess whether serum calcium and magnesium concentrations are causally associated with heart failure. Summary statistics data were collected for seven and six single-nucleotide polymorphisms associated with calcium and magnesium, respectively, from the hitherto largest genome-wide association studies on these minerals. Corresponding summary statistics for genetic associations with heart failure were available from publicly available data based on the UK Biobank study and based on participants of European ancestry. The findings showed that neither serum calcium nor magnesium concentrations were associated with heart failure. In the standard inverse-variance weighted analysis, the odds ratios of heart failure per genetically predicted one standard deviation increase in mineral concentrations were 0.89 (95% confidence interval 0.67–1.17; *p* = 0.41) for serum calcium and 0.89 (95% confidence interval 0.72–1.10; *p* = 0.28) for serum magnesium. Results were robust in sensitivity analyses, including the weighted median and Mendelian randomization Egger analyses. In conclusion, these findings do not support previous findings suggesting a link between serum calcium and magnesium and heart failure, but this study was underpowered to detect weak associations.

## Introduction

Heart failure is a major cause of morbidity and mortality in a large part of the developed world and the global prevalence has been estimated to 37.7 million people (Ziaeian and Fonarow, 2016). The disease is often defined as the clinical syndrome that is manifested when the heart muscle is unable to pump enough blood to meet the body’s needs for oxygen (Tanai and Frantz, 2015). As an attempt to compensate for this impaired heart capacity, the body acts *via* a number of different mechanisms which include enlarging of the heart, increased heart output and narrowing of blood vessels. However, as these measures only are temporary, the patients starts to experience symptoms such as fatigue and breathing problems as the diseases is progressing (Zieian and Fonarow, 2016).

Calcium and magnesium are two minerals that are involved in several physiological processes of the cardiovascular system (Kolte et al., 2014; Reid et al., 2016). Excessive calcium intake has been found to induce hypercalcemia which in turn may result in vascular calcification and thus increased risk of cardiovascular disease (Seely, 1991). On the other hand, magnesium has been suggested to promote vasodilatation partially in a direct manner by acting as a calcium antagonist in smooth muscle cells but also indirectly *via* modulation of endothelial cell function (Kolte et al., 2014). Available evidence indicates that high serum calcium and low serum magnesium concentrations increase the risk of coronary artery disease (Bolland et al., 2011; Del Gobbo et al., 2013; Rohrmann et al., 2016; Larsson et al., 2017; Larsson et al., 2018), a major risk factor for heart failure (Ziaeian and Fonarow, 2016). Yet, few studies has attempted to investigate the associations between these minerals and incidence of heart failure. For calcium, past research shows tendencies towards increased risk of disease (Lutsey et al., 2014). However, findings are not consistent (Van-Hemelrijck et al., 2013; Donneyong et al., 2015). On the other hand and in line with findings for coronary artery disease, an inverse relationship has been suggested for blood levels and dietary intake of magnesium and heart failure incidence (Zhang et al., 2012; Lutsey et al., 2014; Kunutsor et al., 2016; Taveira et al., 2016; Wannamethee et al., 2018). However, due to the limited data available and the fact that most studies, with one exception (Donneyong et al., 2015), were observational, it is uncertain whether the associations are causal and not biased by reverse causality or residual confounding.

In the present study, we applied a two-sample Mendelian randomization design using summary estimates to assess whether serum calcium and magnesium concentrations are causally associated with heart failure. Mendelian randomization is a method that utilizes genetic variants, often in terms of single nucleotide polymorphisms (SNPs), associated with the exposure of interest to estimate the effect on disease outcomes. Due to the random distribution of alleles during gametogenesis, MR has the possibility of evading some of the methodological limitations of other epidemiological study designs, including confounding and reverse causation bias.

## Methods

### SNP Identification and Data Sources

Information on SNPs associated with serum calcium and magnesium concentrations was collected from two meta-analyses of discovery and replication genome-wide association studies (GWASs) of these minerals in European-descent individuals (Meyer et al., 2010; O'Seaghdha et al., 2013). In those GWASs, seven and six SNPs reached genome-wide significance (*p* < 5 × 10^−8^) for serum calcium (among 61,079 participants) and serum magnesium (among 23,829 participants), respectively, and were selected as instrumental variables. The selected SNPs were independent (i.e. not in linkage disequilibrium, r^2^ < 0.001) and explained 0.9% of the variance of calcium concentrations and 1.6% of the variance of magnesium concentrations. Summary statistics data were extracted for each SNP (**Table 1**).

**Table 1:** Characteristics of the single-nucleotide polymorphisms associated with serum calcium and magnesium concentrations and their associations with heart failure.

Mineral	SNP	Chr	Nearby gene	EA	EAF	F-stat	Association with serum calcium or magnesium			Association with heart failure		
							β*	SE	*p* value	β^†^	SE	*p* Value
Calcium	rs1801725	3	*CASR*	T	0.15	299	0.071	0.004	8.9E−86	−0.032	0.027	0.236
Calcium	rs1570669	20	*CYP24A1*	G	0.34	37	0.018	0.003	9.1E−12	0.014	0.018	0.457
Calcium	rs1550532	2	*DGKD*	C	0.31	37	0.018	0.003	8.2E−11	0.004	0.018	0.829
Calcium	rs7481584	11	*CARS*	G	0.70	37	0.018	0.003	1.2E−10	0.003	0.018	0.888
Calcium	rs780094	2	*GCKR*	T	0.42	37	0.017	0.003	1.3E−10	0.008	0.017	0.652
Calcium	rs7336933	13	*DGKH/KIAA0564*	G	0.85	31	0.022	0.004	9.1E−10	−0.013	0.024	0.594
Calcium	rs10491003	10	*GATA3*	T	0.09	31	0.027	0.005	4.8E−09	−0.030	0.031	0.344
Magnesium	rs4072037	1	*MUC1*	T	0.53	136	0.010	0.001	2.1E−36	0.006	0.017	0.744
Magnesium	rs7965584^‡^	12	*ATP2B1*	A	0.71	60	0.007	0.001	1.1E−16	−0.021	0.020	0.286
Magnesium	rs3925584	11	*DCDC5*	T	0.55	60	0.006	0.001	5.2E−16	0.009	0.017	0.607
Magnesium	rs11144134	9	*TRPM6*	C	0.08	55	0.011	0.001	8.2E−15	−0.022	0.032	0.505
Magnesium	rs13146355	4	*SHROOM3*	A	0.44	45	0.005	0.001	6.3E−13	−0.052	0.018	0.003
Magnesium	rs448378	3	*MDS1*	A	0.53	31	0.004	0.001	1.3E−08	0.003	0.017	0.864

Chr, chromosome; EA, effect allele (i.e., the allele associated with higher calcium or magnesium concentrations; F-stat, F-statistic; SE, standard error.

*β coefficient of the calcium- or magnesium-increasing allele on serum concentrations of calcium (in mg/dl) and magnesium (in mmol/L).

^†^β coefficient of the mineral-increasing allele on the log odds ratio of heart failure.

^‡^A proxy SNP (rs10858938) associated with the specified SNP (pairwise r^2^ = 0.95) was used in the heart failure dataset. The effect allele for the proxy SNP is G.

Following SNP identification, publicly available summary statistics data on SNP–heart failure associations were obtained from a discovery GWAS on heart failure in the UK Biobank study (Aragam et al., 2018) and accessed through the Broad Institute Cardiovascular Disease Knowledge Portal (http://broadcvdi.org). In the UK Biobank study, all-cause heart failure was defined as either self-reported heart failure/pulmonary edema or cardiomyopathy or an international classification of disease code indicative of heart/ventricular failure or a cardiomyopathy of any cause (Aragam et al., 2018). Individuals with left ventricular dysfunction and without coronary artery disease was defined as having non-ischemic cardiomyopathy (Aragam et al., 2018). The heart failure dataset included summary statistics data based on 394,156 participants (6,504 all-cause heart failure cases and 387,652 non-cases) of European ancestry. The analyses of the underlying studies included in the GWASs of calcium, magnesium, and heart failure were adjusted for age, sex and, if needed, study-specific covariates (e.g., principal components of ancestry and study center). There was no overlap between individuals included in the GWASs on the minerals and those included in the GWAS on heart failure.

### Assessment of Pleiotropy

We searched the human genotype-phenotype association database PhenoScanner V2 (Kamat et al., 2019) for pleiotropic associations of the instrumental variables with potential confounders. The following SNPs were identified as pleiotropic with potential confounders: rs780094 near *GCKR* which is associated with the metabolic syndrome and its components (blood lipids, fasting glucose and insulin concentrations, and type 2 diabetes); rs3925584 near *DCDC5* and rs13146355 near *SHROOM3* which are associated with kidney function; and rs448378 near *MDS1* which is associated with blood pressure. The association with blood pressure was considered to represent vertical (mediated) pleiotropy because magnesium supplementation has been shown to reduce blood pressure in randomized controlled trials (Zhang et al., 2016). The other associations may represent horizontal pleiotropy and the corresponding SNPs were excluded in sensitivity analyses.

### Statistical Analysis

**Figure 1:**
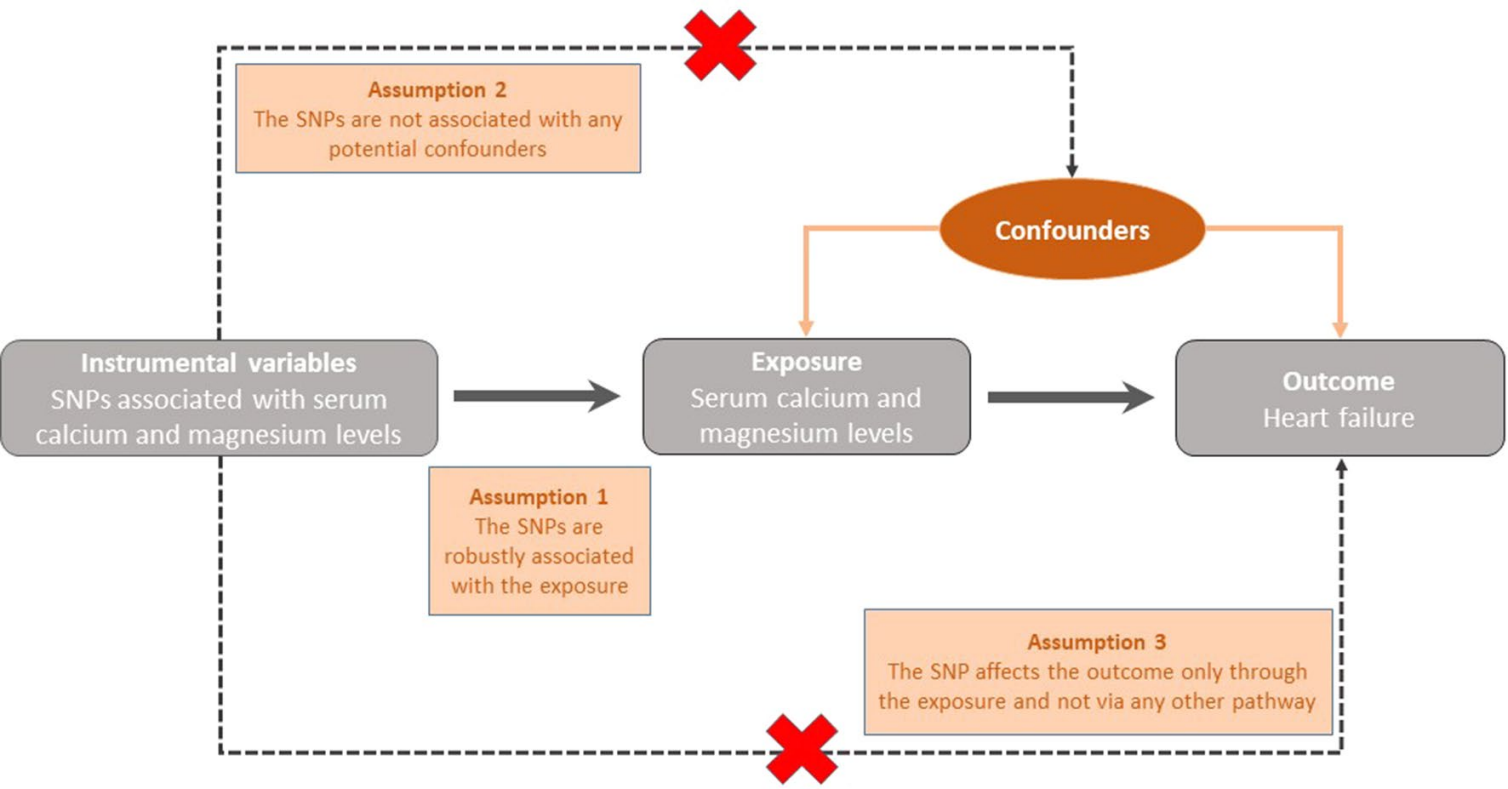
Graphical representation of the key assumptions underlying the Mendelian Randomization study design. SNP; Single nucleotide polymorphism.

The primary analyses of causal associations of instrument variables for serum calcium and magnesium with all-cause heart failure were performed using the standard inverse variance weighted method (Burgess et al., 2017). By using this method, the causal effect of an exposure on an outcome is estimated as the ratio (Wald estimate) of the SNP-outcome association and the SNP-exposure association. The inverse weighted variance method provides a reliable estimate of the causal effect of the exposure on the disease outcome if the following assumptions are valid for the instrument variables: 1) the SNPs are robustly associated with the exposure of interest 2) the SNPs are not associated with any potential confounders of the exposure-outcome relationship and 3) the SNP only affects the outcome through the exposure and not *via* any other causal pathway (**Figure 1**) (Smith and Ebrahim, 2003; Grover et al., 2017). Secondly, we performed sensitivity analyses using the weighted median (Burgess et al., 2017), weighted mode-based estimate (Hartwig et al., 2017) and MR-Egger methods (Burgess et al., 2017). The weighted median method provides a reliable effect estimate when at least 50% of the weight in the analysis comes from valid instrumental variables. The MR-Egger method is used for detection of and adjustment for directional pleiotropy. In a secondary analysis, we used the inverse-variance weighted method to analyze the associations between genetically predicted serum calcium and magnesium concentrations and nonischemic cardiomyopathy. All odds ratios (OR) were scaled to reflect a one standard deviation (SD) increment of serum calcium and magnesium concentrations (0.5 mg/dl and 0.1 mmol/dl for calcium and magnesium, respectively). The SD corresponded to the rounded mean SD of serum concentrations of calcium and magnesium in the cohorts included in the GWASs of these minerals (Meyer et al., 2010; O'Seaghdha et al., 2013). The statistical analysis was carried out using Stata (StataCorp, College Station, TX) and the mrrboust package (Spiller et al., 2018). All tests were two sided and the level of significance was set to a Bonferroni-corrected threshold of *p <*0.01 (correcting for two exposures and two outcomes). Statistical power was estimated by using the method by Brion et al. (Brion et al., 2013). We had 80% power to detect an OR of 1.37 (or 0.63) for serum calcium and 0.72 (or 1.28) for serum magnesium concentrations. However, because the number of heart failure cases was relatively small, we also used 97 SNPs associated with body mass index (BMI) (Locke et al., 2015) as a positive control and made post-hoc power analyses to assess the statistical power of the present study.

## Results

**Figure 2:**
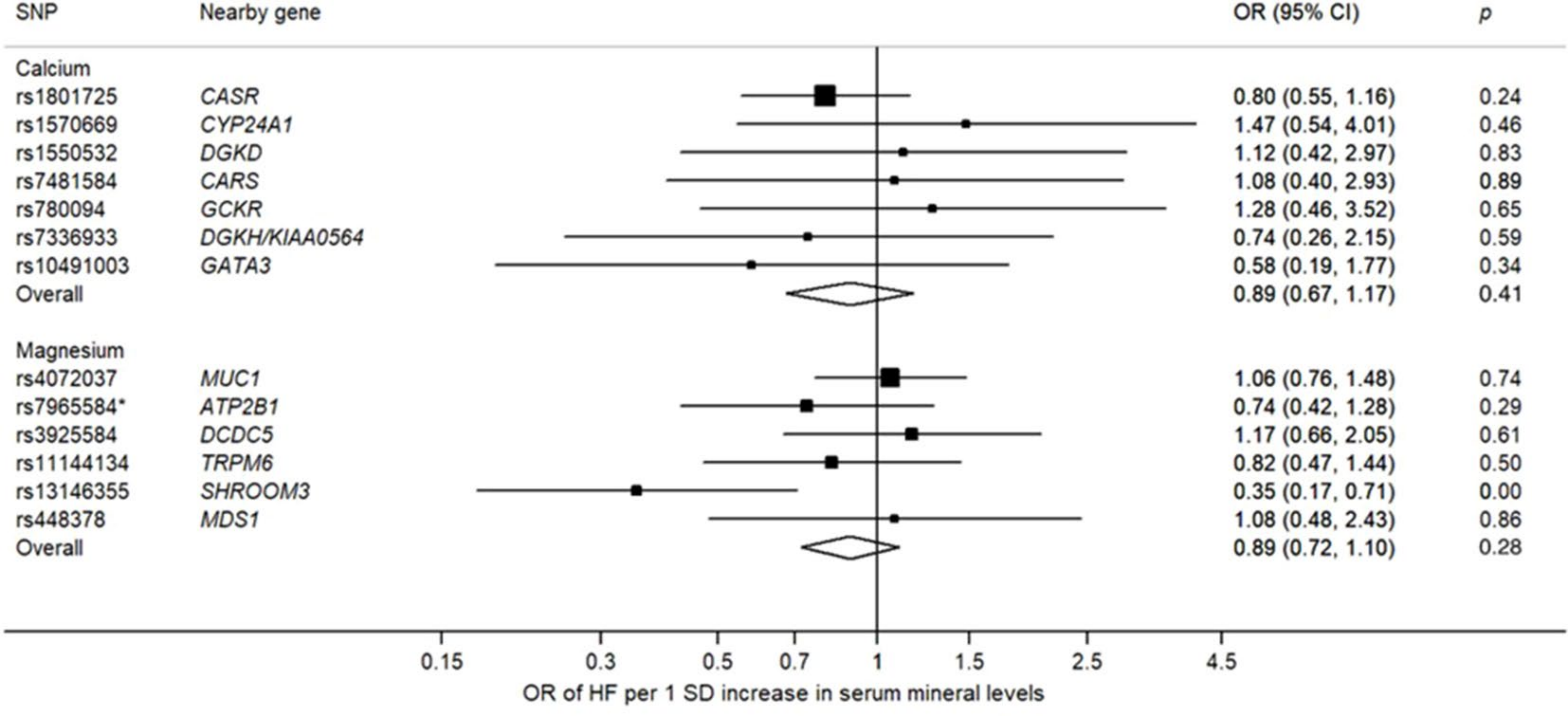
Associations of genetically predicted one standard deviation increase of serum calcium and magnesium concentrations with heart failure. One standard deviation corresponds to 0.5 mg/dl and 0.1 mmol/dl for calcium and magnesium, respectively. Overall refers to the overall estimate generated by the inverse variance weighted method, combining all SNPs. CI, confidence interval; OR, odds ratio; SD, standard deviation; SNP, single-nucleotide polymorphism. *A proxy SNP (rs10858938) associated with the specified SNP (pairwise r^2^ = 0.95) was used in the heart failure dataset.

**Figure 3:**
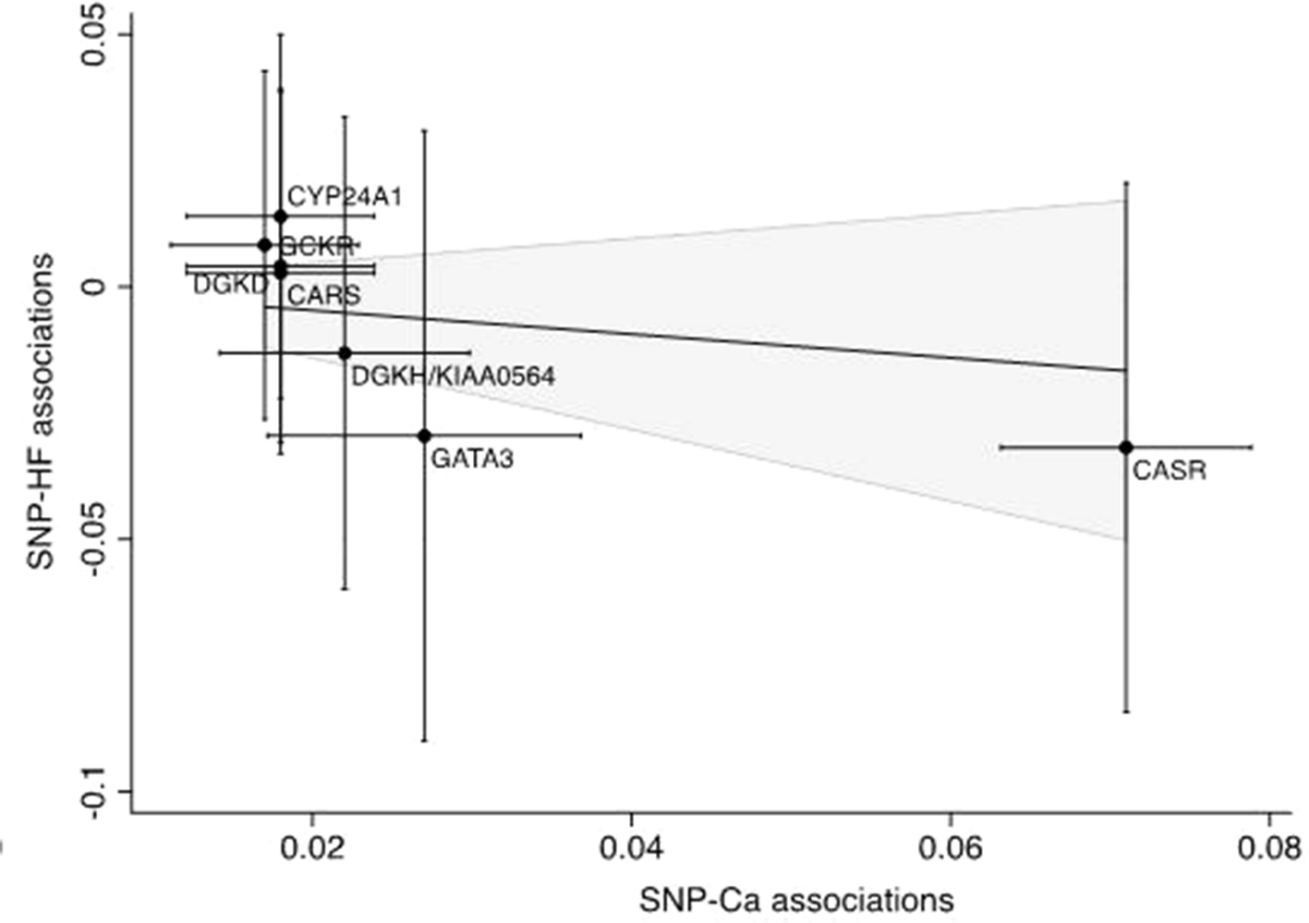
Scatter plot of the summary statistics estimates for calcium and heart failure. The solid line represents the inverse-variance weighted estimate. SNP, single-nucleotide polymorphism; HF, heart failure; Ca, Calcium.

**Figure 4:**
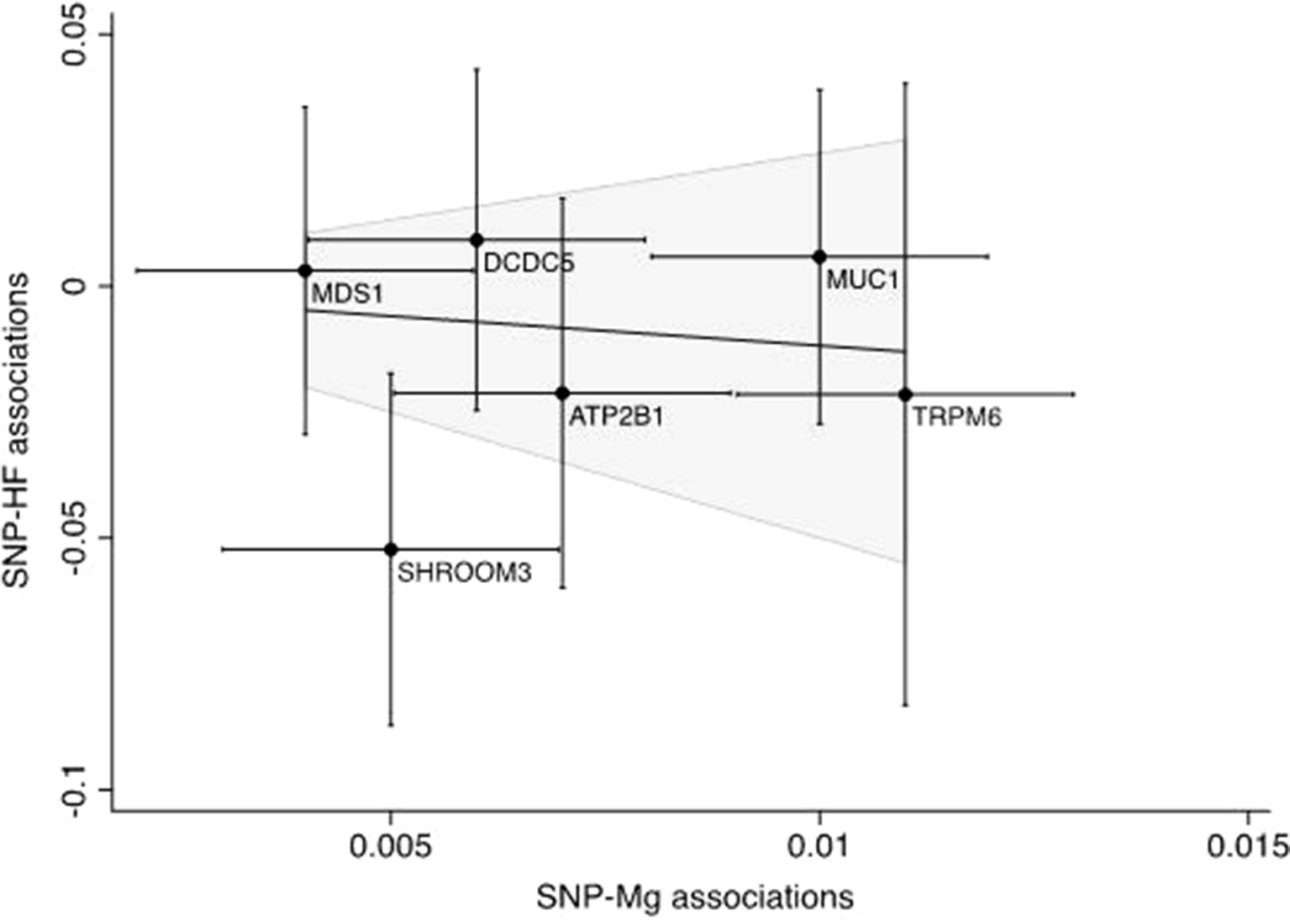
Scatter plot of the summary statistics estimates for magnesium and heart failure. The solid line represents the inverse-variance weighted estimate. SNP, single-nucleotide polymorphism; HF, heart failure, Mg, magnesium.

The associations of genetically predicted serum calcium and magnesium concentrations with all-cause heart failure based on the inverse variance weighted method are presented in **Figures 2**–**4**. None of the calcium-related SNPs nor the overall estimate, combining all SNPs, were associated with heart failure (OR 0.89 95% CI 0.67–1.17; *p* = 0.41), and there was no heterogeneity among SNPs (*p* = 0.83). Of the six magnesium-related SNPs, rs12146355 near *SHROOM3* was associated with heart failure (*p* = 0.003). However, the association with heart failure across all six SNPs did not reach statistical significance (OR 0.89 95% CI 0.72–1.10; *p* = 0.28) but there was suggestive evidence of heterogeneity among SNPs (*p* = 0.09). After exclusion of the SNP in *SHROOM3*, the OR was 0.98 (95% CI 0.78–1.22) and there was no heterogeneity between SNPs (*p* = 0.73). The null associations of serum calcium and magnesium concentrations with heart failure remained after exclusion of SNPs associated with the metabolic syndrome and its components (rs780094) and kidney function (rs3925584 and rs13146355). The null associations also remained when using the weighted median method; the ORs were 0.87 (95% CI 0.62–1.22) for calcium and 0.98 (95% CI 0.75–1.29) for magnesium. In analysis using the weighted mode-based estimate, the ORs were 0.81 (95% CI 0.57–1.16) for calcium and 1.05 (95% CI 0.78–1.41) for magnesium. The MR-Egger regression analysis did not reveal any signs of directional pleiotropy for calcium (intercept 0.017; 95% CI −0.012 to 0.046; *p* = 0.24) or magnesium (intercept −0.023; 95% CI −0.088 to 0.043; *p* = 0.50). Moreover, genetically predicted serum calcium and magnesium concentrations were not associated with non-ischemic cardiomyopathy (n = 1816 cases); the ORs were 1.02 (95% CI 0.61–1.69; *p* = 0.48) for calcium and 0.80 (95% CI 0.53–1.19; *p* = 0.67) for magnesium. As a positive control, we used BMI, which was statistically significantly positively associated with heart failure. The OR was 1.53 (95% CI 1.32–1.77; *p* = 2.1 × 10^−8^) per one SD increase in genetically predicted BMI. The post-hoc power analyses suggested that we had about 13% and 20% power to detect an OR of 0.89 for calcium and magnesium, respectively.

## Discussion

The overall results of this MR study did not find any evidence for a causal association between minerals and heart failure and thus do not support findings from previous observational studies, suggesting a positive association between serum calcium and heart failure risk (Lutsey et al., 2014) as well as an inverse association between serum magnesium and heart failure risk (Zhang et al., 2012; Lutsey et al., 2014; Kunutsor et al., 2016; Taveira et al., 2016; Wannamethee et al., 2018). However, given that previous studies are scarce and findings are limited by potential residual confounding and reverse causality, caution must be applied when interpreting these data. On the other hand, and in line with our results, heart failure was neither associated with baseline blood calcium in an observational study (Van-Hemelrijck et al., 2013), nor affected by calcium supplementation in a randomized controlled trial (Donneyong et al., 2015).

Of the six loci associated with magnesium, *SHROOM3* was statistically significantly inversely associated with heart failure. The SNP in this locus is associated with decreased glomerular filtration rate and therefore may increase serum magnesium concentrations *via* decreased renal filtration and excretion of the ion. However, as there was no consistent association with any of the other five SNPs with heart failure, it is not likely that there is a causal association between serum magnesium concentrations and heart failure.

The major strength of this study is the MR design, which reduces the risk of bias from confounding factors and reverse causality. The most important limitation is the low statistical power resulting from the relatively small number of heart failure cases in the outcome data set and the small proportion of variance in serum calcium and magnesium concentrations explained by the instruments. Together, it is possible that these factors might have hampered the detection of a potential relationship between minerals and heart failure. On the other hand, the number of heart failure cases was sufficient to detect a statistically significant association between the positive control, BMI, and heart failure. Moreover, previous studies of the MR design using the same instruments have demonstrated statistically significant positive and inverse associations of serum concentrations of calcium and magnesium, respectively, with other cardiovascular diseases (Larsson et al., 2017; Larsson et al., 2018; Larsson et al., 2019a; Larsson et al., 2019b). However, the post-hoc power analysis suggested that we only had ∼20% power to detect an OR of 0.89 for magnesium and 13% power to detect an OR of 0.89 for calcium. Thus, we cannot rule out that there may be an effect of low magnitude for calcium and magnesium on heart failure which this study was unable to detect. Thus, we call for cautious interpretation of these findings.

Another issue that has to be addressed in MR studies is pleiotropy, which can be either vertical or horizontal. Vertical pleiotropy occurs if genetic variants associates with other traits downstream the main trait and thus mediates the effect of the former. This type of pleiotropy does not violate the MR assumptions and does not bias the MR effect estimates. Horizontal pleiotropy occurs if SNPs have an effect on the outcome through another trait or pathway than the one under investigation. If the horizonal pleiotropy is directional, all traits have similar effects on the outcome and will lead to an exaggerated MR estimate. If horizonal pleiotropy is balanced, the different traits will have dissimilar effects on the outcome and the association will instead be biased towards the null. In this study, we assessed occurrence of direction horizontal pleiotropy by applying MR-Egger regression. The results of the analysis did not reveal any signs of pleiotropic effects. However, this method has low power, especially when the number of SNPs are limited. For this reason, we also performed a PhenoScanner search through which we identified four SNPs that had pleiotropic associations with potential confounders or intermediates. However, the null associations of serum calcium and magnesium concentrations with heart failure remained after exclusion of those SNPs. A final limitation of our study is that we did not have access to individual-level data and therefore could not examine potential non-linear associations of serum calcium and magnesium concentrations with heart failure risk.

## Conclusions

The present study based on the MR design did not find any evidence for a causal association between minerals and heart failure. Thus we were unable to confirm the positive and inverse association of serum calcium and magnesium, respectively, with heart failure risk that have been observed in previous observational studies. Given the scarcity of studies addressing the research question and the inconclusive findings, further research on the possible causal role of calcium and magnesium for heart failure are needed.

## Data Availability Statement

Publicly available datasets were analyzed in this study. This data can be found here: https://journals.plos.org/plosgenetics/article?id=10.1371/journal.pgen.1003796, https://journals.plos.org/plosgenetics/article?id=10.1371/journal.pgen.1001045, http://www.broadcvdi.org/.

## Author Contributions

EH and SL designed the research and performed the statistical analyses. EH wrote the first draft of the manuscript. EH, AÅ, and SL interpreted the data, made critical revisions of the manuscript, and approved the final manuscript.

## Funding

This work was supported by the Swedish Research Council.

## Conflict of Interest

The authors declare that the research was conducted in the absence of any commercial or financial relationships that could be construed as a potential conflict of interest.
